# Methods for Multiplex Template Sampling in Digital PCR Assays

**DOI:** 10.1371/journal.pone.0098341

**Published:** 2014-05-22

**Authors:** Oleh I. Petriv, Kevin A. Heyries, Michael VanInsberghe, David Walker, Carl L. Hansen

**Affiliations:** 1 Centre for High-Throughput Biology, University of British Columbia, Vancouver, British Columbia, Canada; 2 Department of Physics and Astronomy, University of British Columbia, Vancouver, British Columbia, Canada; Naval Research Laboratory, United States of America

## Abstract

The efficient use of digital PCR (dPCR) for precision copy number analysis requires high concentrations of target molecules that may be difficult or impossible to obtain from clinical samples. To solve this problem we present a strategy, called Multiplex Template Sampling (MTS), that effectively increases template concentrations by detecting multiple regions of fragmented target molecules. Three alternative assay approaches are presented for implementing MTS analysis of chromosome 21, providing a 10-fold concentration enhancement while preserving assay precision.

## Introduction

Digital PCR (dPCR) is a precise DNA quantification technique based on the isolation of single template molecules at limiting dilution followed by amplification and end-point detection [1]. The recent availability of dPCR instrumentation, including droplet formats and microfluidic formats [2], [3], [4], [5], [6], [7], has made this method broadly available and has opened new opportunities in basic and clinical research. dPCR has important analytical advantages for diagnostic testing, including precise and absolute measurement of nucleic acid biomarkers, reliable single molecule sensitivity, enhanced specificity for the detection of rare alleles within a background of similar sequences, and ease of assay standardization and data interpretation.

One important application of dPCR is the detection and accurate measurement of small copy number variations in cell free DNA that may arise through gene amplifications or aneuploidy within a fraction of the DNA pool [8], [9]. When well-optimized, the precision of a dPCR measurement is limited only by binomial noise and is determined by the total number of PCR sub-reactions performed for each measurement. In previous work [2] we have shown that arrays of 1,000,000 reactions allow for the reliable detection of allelic imbalances as small as 1%. However, achieving this precision requires that the array be loaded with template at a sufficient concentration to ensure that a substantial number of reactions have target molecules, with the optimal concentration of target molecules being approximately one template molecule per PCR sub-reaction. This presents a practical problem in clinical testing since the plasma fraction obtained from a typical 5 mL blood sample will contain only ∼25,000 genome copies (approximately 5000/mL) [10], negating the advantages of dPCR arrays larger than ∼50,000 reactors. In addition, the precision of measurement is fundamentally limited by sampling noise in the original sample; assuming a Poisson distribution of template molecules at an average of 25,000 copies per sample, this corresponds to a standard deviation (σ) of 0.6% and a limit of detection of approximately 3% (based on 5σ separation). Furthermore, performing dPCR analysis requires that the DNA be concentrated into a microliter-scale volume, presenting a formidable technical challenge. One approach to increasing the concentration of template molecules is to perform PCR specific target amplification prior to dPCR analysis [11]. While this approach is attractive for some dPCR applications, including the detection of rare SNVs, it does not address the issue of sampling noise, and may introduce additional biases [4] that if not well-characterized and controlled can further limit precision.

Here we present an alternative strategy, called Multiplex Template Sampling (MTS), to effectively increase template concentrations in dPCR analysis. MTS exploits the fact that a large template molecule, such as a chromosome, is highly fragmented in plasma. Simultaneous amplification and detection of multiple regions in a single fluorescent channel thus provides a means to enhance the effective copy number and thereby increase the precision and sensitivity of dPCR analysis.

**Figure 1:**
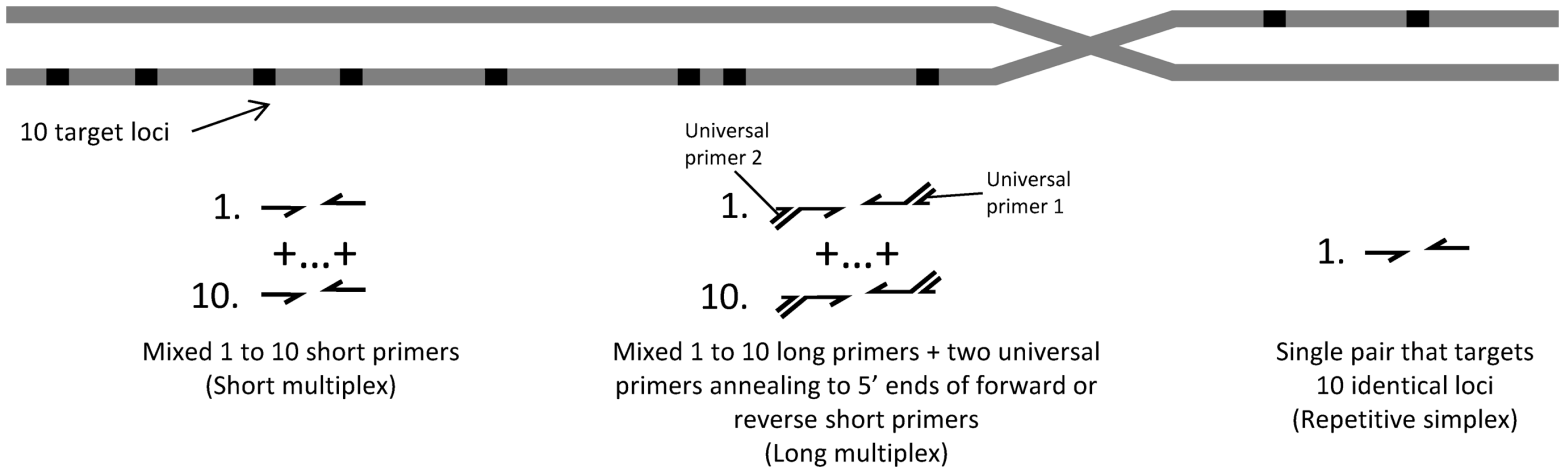
Multiplex Template Sampling (MTS) strategies. In the first, “short multiplex”, the sampling rate is raised by ten primer pairs in a multiplexed PCR with a common fluorescent probe for detection. The second method, “long multiplex”, is similar, but loci-specific primers are longer and we append a universal sequence to them. A third method, “repetitive simplex”, uses a single PCR assay designed to target chromosome-specific repetitive sequences.

As a demonstration of the MTS method we show that the effective concentration of chromosome 21 in normal human genomic DNA may be increased linearly by increasing the number of assayed loci, ultimately achieving a 10-fold enhancement. Here we present three alternative assay strategies for MTS and compare their performance (Figure 1). In the first, the sampling rate is raised by simply using multiple primer pairs in a multiplexed PCR with a common fluorescent probe used for detection (hereafter referred to as “short multiplex”). The second method improves the performance of this approach by appending a universal sequence to a long loci-specific primer (hereafter referred to as “long multiplex”). Finally, we present a third method that uses a single PCR assay designed to target chromosome-specific repetitive sequences (hereafter referred to as “repetitive simplex”). Using each of these three approaches we demonstrate an effective ten-fold increase in template concentration which could be applied to dPCR measurements of chromosome imbalance.

## Materials and Methods

### PCR Reactions

#### Oligonucleotides and fluorescent probes

For the short multiplex design ten primer pairs (18–25 bp long) were designed to anneal at ten different loci located along Human chromosome 21 (bold sequences 21.1 to 21.10, Table 1). These loci were selected to contain a common 8 base pair sequence that is complementary to a single FAM-labeled locked nucleic acid (LNA) probe (probe #36 from the Roche Universal Probe Library, Cat. # 04687949001). Amplicons were designed using a commercially available on-line service (Universal Probe Library Assay Design Center; Roche), generating alternative flanking primers that span the probe #36 site (GGAGCCAG). In addition, to normalize the amount of total DNA loaded in each experiment, we designed a single pair of primers and a single fluorescent hydrolysis probe (CalOrange^560^-CAGCCACACATTCCCTCCCCGTCC-BHQ2; Biosearch Technologies Inc.) to target RNAse P, a single copy gene located on chromosome 6. For the long multiplex design the same primers were used with an additional common sequence appended to the 5′ end. Primer sequences for the short multiplex, long multiplex, and repetitive multiplex designs are included in Table 1.

**Table 1:** Pairs of long and short oligonucleotides used in multiplexed PCR reactions.

Target	Primer Sequences*
**21.1**	(U1)-**TGTTAGGCATCCACACAAGC**GTGCTGTGCCTGCC
	(U2)-CAGCATGACCACCACA**CATTCAGGACTCCAGCTCAGA**CCTGA
**21.2**	(U1)-**TGTGTCAGAGAACCGTGGTCCTGC**CTGAGGCCA
	(U2)-CTGTG**GCAGCTGGCATTCTCCTC**CGGGA
**21.3**	(U1)-GGGAAGGAGAAAAG**AGCTGCAACCCTTCTGGAC**ATCTAGACCTCAGAGCTTC
	(U2)-CAGCACCTGTGAAGCC**CCAAAGGGGGTGTTACATCA**TGTCAC
**21.4**	(U1)-AGCGGAAGA**ACTGGGCTGTATCAGCCATC**TTACAAGACCCTCC
	(U2)-CCTCGCTCCTAATTTTTAAATAC**CATCTTTCTGTTTACCTTCTTGTCC**TTAAGGAAAACGCA
**21.5**	(U1)-CCATGCTGCAGCTC**CTGCAGGACAAGGACACCTAC**
	(U2)-**TTCAGGAACTTCCGCTTGTC**GCTGGTGTCGCTCC
**21.6**	(U1)-TGGATGATCTGTCCCTTATTCAA**GGGCTTCCCTACTTAGATCTGG**CAGGA
	(U2)-AGTTCCTTGTAC**AAAGGAACAGGGGGAGGA**GCTCCCTCA
**21.7**	(U1)-CCTGCTCATTCATTAACATGATG**TGTGTTTATGGAGAGCTGACTTG**TACCAGGCACGGTTC
	(U2)-GTGTAGCTGTCTGTT**TACCTGTCACCGCAACCAG**CCTGTGT
**21.8**	(U1)-GGAAGCAACAGAGGAGAAAGGCAGGATATTTGG**TGTCTGCTCACATTGGGAAA**
	(U2)-TGTAGCCCTGCAGACAGA**CATCCAGCCTTCTCTGGACT**TAACC
**21.9**	(U1)-GGCAGCCTCG**GGTGGCATTCTGCACGTT**
	(U2)-**ACAGGAAGTTAGACCCAAGCTG**CGCCGACACTCACCT
**21.10**	(U1)-TGGGACACCTAAAAGTTAAGAAAGGTGC**CCCAGGGACACAGCTAGTAAGT**GG
	(U2)-GAGCAC**AGATTCTGCCGCCATCCT**GCCCAGATTTCC
**6.1**	(U1)-**CCTGGCATGACGTGTGTCTG**CGTCTGTCCACGT
	(U2)-GGATCTCAGTGGAATGGAGA**TGAGCAGAGTAGAAAGAGCACATC**CCACAGGGC

Short primers are shown in bold. The first ten pairs target different loci on chromosome 21. The last pair targets RNAse P on chromosome 6. The oligonucleotides used for long multiplex experiments are full sequences listed in the table, with the addition of the universal sequence GACTGACTGCGTAGGTATTATCG (designated as U1 in the table) for forward primers and CACAGGAAACAGCTATGACC (designated as U2 in the table) for the reverse at 5′ end of the primers. The primers used for the repetitive simplex experiments target ten loci on chromosome 21 and were CCTGGTCTGCACCCCAGTG and GTGCAGGAGCTGGTGCAG, and were used with probe #74 (Cat. #04688970001) from the Roche Universal Probe Library which anneals to the CTGCTGCCC motif.

* Universal sequences on 5′ ends of long pairs are not shown.

#### Composition

All PCR reactions were prepared in 10 µL volumes consisting of 5 µL of 2x TaqMan Fast Universal PCR Master Mix (Applied Biosystems, USA), 1 µL of 1% Tween-20 (Sigma), 0.5 µL of 50 mM MgCl_2_, 0.3 µL of dNTPs mix (100 mM), 0.2 µL of Platinum Taq polymerase (5 U/µL, Invitrogen, USA), 0.1 µL of FAM fluorescent probe (10 µM stock), 0.1 µL of CalOrange fluorescent probe (100 µM stock), genomic DNA (Biochain #D1234-152) to desired concentration and H_2_O to 10 µL. Fragmentation of genomic DNA was facilitated by heating to 95°C for 10 min. Digital arrays imaged using the confocal laser scanner were supplemented with 500 nM Quasar670 (Biosearch Technologies Inc). Primer combinations and concentrations were changed depending on the assay as described below.

#### Primer Combinations

There were four oligonucleotide combinations used: a) In order to initially validate each primer pair, each of the short and long pairs (21.1–21.10) were tested independently in simplex at a concentration of 250 nM each. Additionally, pair 6.1 was included in each reaction to normalize DNA loading concentration. b) Short pairs 21.1 to 21.10 were sequentially added to pair 6.1 at a final concentration of 250 nM each for short multiplexing experiments. c) For long multiplexing experiments, long pairs 21.1 to 21.10 were sequentially added to pair 6.1 at a final concentration of 50 nM each. The two universal primers were also added at a final concentration of 1 µM each. d) The repetitive sequence pair was added to short pair 6.1, all at a concentration of 1 µM each. Additionally, genomic DNA for this combination was pre-treated with BglII endonuclease (New England BioLabs) in the recommended buffer for 10 min at 37°C.

#### Thermal protocol

The four thermal protocols used for the different assays are listed in Table 2.

**Table 2:** Four thermal protocols used with different PCR reaction and chip designs.

Protocol 1			Protocol 2			Protocol 3			Protocol 4					
Time,sec	Temp., °C	Cyc.	Time, sec	Temp., °C	Cyc.	Time, sec	Temp., °C	Cyc.	Time, sec		Temp., °C			Cycles
***Hot Start***			***Hot Start***			***Hot Start***			***Hot Start***					
180	95	1	180	95	1	30	95	1	20		90			1
***Denaturation***			***Denaturation***			***Denaturation***			***Denaturation***					
15	95	all	15	95	all	15	95	all	5		92			all
***Extension***			***Extension***			***Extension***			***Extension***					
120	72	2	120	75	2	30	62	3	***Long primers***					Short primers*
60	71	2	120	74	4	30	61	3	60	72			1	
60	70	2	120	73	4	30	60	2	60	71			1	
40	59	1	100	72	4	30	59	2	40	70			1	
30	58	1	30	68	4	30	58	6	40	68			1	
20	57	1	20	66	3	15	56	34	40	66			1	
15	56	31	20	64	3				35	64			1	
			20	62	3				30			62		3
			20	60	3				30			61		3
			20	58	3				30			60		2
			15	56	7				30			59		2
									15			58		22

* Initial cycles done only in presence of long primers.

#### Microfluidic chips

The microfluidic chips were fabricated using standard multilayer soft lithography as described in references [2] and [12]. We used two chip formats: 2-nL, with ten arrays of 765×2-nL PCR chambers and 100-pL, with ten arrays of 10,000×100-pL PCR chambers. After the 2-nL chips were loaded with the appropriate reagents and valves locked, they were placed in a prototype version of the Biomark Instrument (Fluigim Corp.) [13] for thermocycling. After each PCR cycle, fluorescent images from the devices were taken. For dPCR experiments in high-density devices which do not have microvalves, each sample lane (10,000×100 pL chambers) was loaded and isolated using fluorinated oil (FC40, Sigma) as previously described [2]. These chips were cycled at flatbed thermocycler (BioRad DNA Engine PTC-200) and imaged using a confocal laser scanner (Wellscope, Biomedical Photometrics).

#### Digital PCR data analysis

For 2-nL format chips, data was analyzed using custom scripts written in MATLAB (MathWorks). Real-time curves were generated from these data and numbers of positive chambers in the array counted. For 100-pL format chips, custom image analysis software, written in C, was used for automated segmentation of chambers (using the passive dye) and counting of positive chambers. A best estimate of molecule numbers *λ* was derived from the number of positive chambers *x* in an array with *N* chambers based on the binomial distribution: 
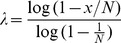
.

## Results

### Long and short primers for multiple DNA sampling

#### Assay validation

**Figure 2:**
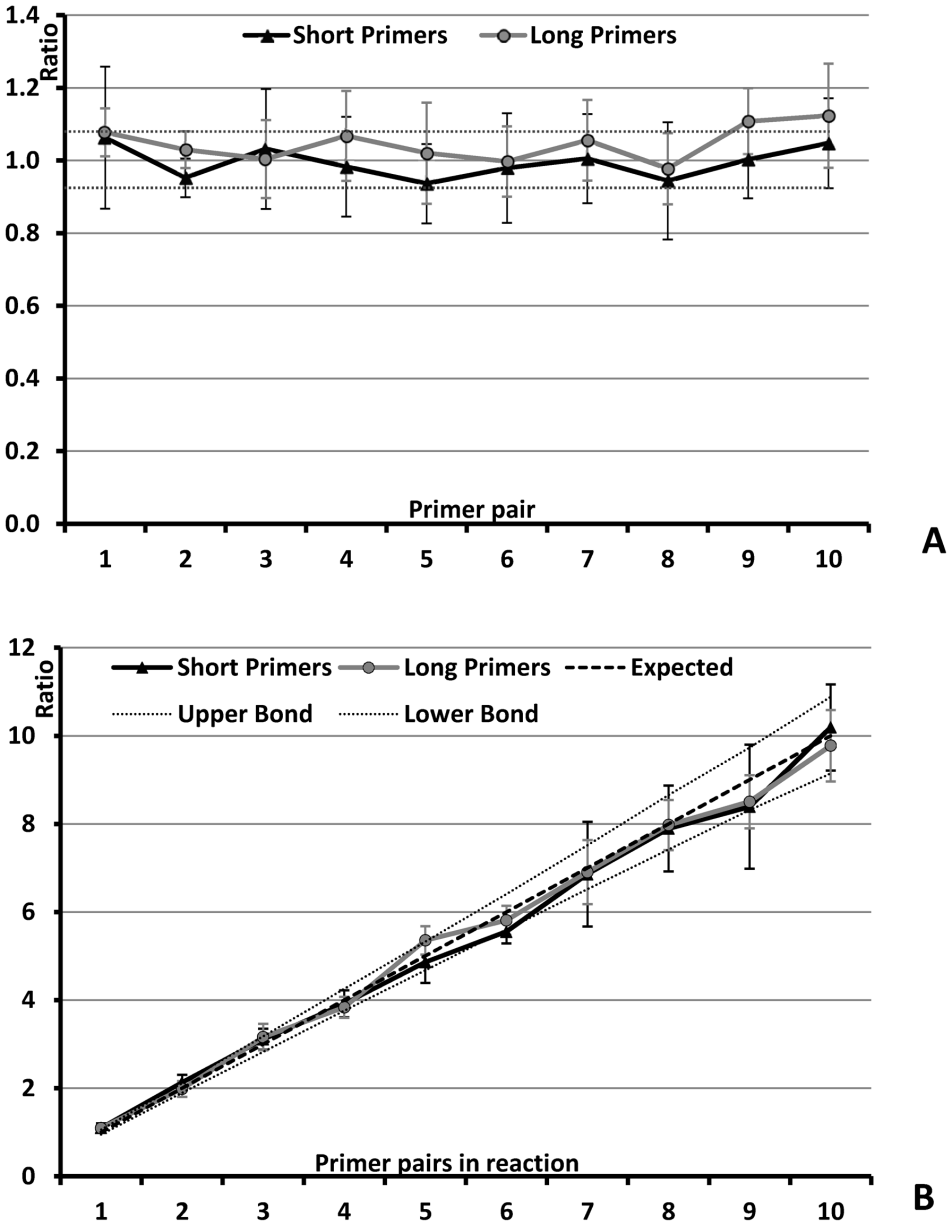
Assessment of chromosome ratio using “short” and “long” multiplexing strategies. (**A**) Mean ratio (n = 5) between chromosomes 21 and 6 in normal human adult male DNA as measured by dPCR using ten different primer pairs each specific to one locus on chromosome 21. (**B**) Mean ratio (n = 5) between chromosome 21 and chromosome 6 as measured by dPCR using multiplexed reactions with one to ten primer pairs in the PCR mix. Reaction volumes are 2 nL, multiplexing level (horizontal axis) increases from 1× to 10×. Two approaches compared: with short primers and long primers plus a pair of universal primers. Upper and lower boundaries of 95% confidence intervals [22] are shown with dashed lines.

Using 2 nL digital PCR arrays, we measured the relative ratio between each of the chromosome 21 assays and the reference chromosome 6 assay (Figure 2A). In all cases no template controls (NTCs) resulted in no positive chambers (data not shown). The ratio of chromosome 21 to chromosome 6 that was obtained for all primer pairs was found to be close to 1∶1 as expected. Thermal protocols used for validation were Protocol 1 for short primers and Protocol 2 for long primers (Table 2).

#### Direct multiplexing with short primers

Using 2 nL digital PCR arrays, we performed ten dPCR reactions containing a primer pair targeting chromosome 6 and varying levels of chromosome 21 multiplexing, targeting between one and ten loci (mean distance 2.1 Mbp from each other) (Figure 2B). The ratio of chromosome 21 to chromosome 6 pairs was found to increase proportionally to the multiplexing level with all measured ratios falling within a 95% confidence interval constructed around the expected ratios. The NTCs in all experiments did not generate positive hits (data not shown). However, we observed an increase in background fluorescence and decrease in signal to noise ratio at higher multiplexing, resulting in difficulties when discriminating positive reactions. We hypothesized that this observation was due to an increase in nonspecific amplification arising from high total primer concentrations and resulting primer-primer interactions. This problem was solved by implementing a touch-down PCR protocol and lowering the primer concentration to 250 nM per primer, bringing the total concentration of primers to 5.5 µM (22×250 nM). Although higher primer concentrations increase the amount of non-specific side reactions, this effect appears to be mitigated by improved specific in early cycles (due to higher annealing temperatures) and sustaining amplification for longer to achieve sufficient signal to noise ratio.

#### Multiplexing with long primers and universal extensions

**Figure 3:**
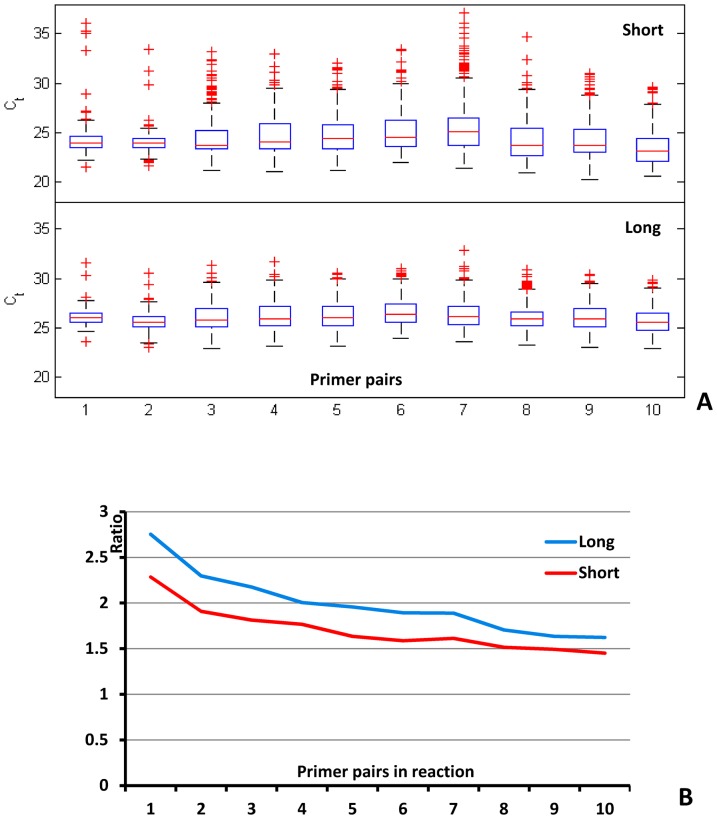
dPCR quality comparison between “short” and “long” multiplexing strategies. (**A**) Boxplots for comparison of Ct values (vertical axis) distribution in 2 nL PCR compartments filled with variable multiplexing level (horizontal axis) primer mixes. Upper panel – PCR mixes supplemented with short primers. Lower panel – PCR mixes supplemented with combination of long and plus a pair of universal primers. Red crosses denote outliers that are larger than the 75^th^ percentile plus 1.5× the interquartile range or smaller than the 25^th^ percentile minus 1.5× the interquartile range. This corresponds to approximately ±2.7σ and 99.3% coverage assuming that the data are normally distributed. (**B**) The ratio between mean fluorescence in positive chambers in dPCR arrays and background fluorescence in negative chambers.

While reducing the total concentration of primer pairs should reduce nonspecific amplification, low primer concentrations do not support sufficient amplification for detection of positive wells. Thus, we designed a long multiplexing strategy to separate these two requirements, allowing for initial priming off low concentrations of loci-specific primers, followed by “developing” these reactions using amplification of all sites via a common sequence appended to the 5′ end of each loci-specific primer (the universal sequences U1 and U2 in Table 1). This strategy was tested using two-stage PCR approach that uses high annealing temperature to initiate amplification of target loci, followed by lower annealing temperatures to develop these reactions. The starting annealing temperature in the thermal protocol was raised by 3°C and the time of annealing in initial cycles was extended (Thermal Protocol 2, Table 2). Using this protocol and specific primers concentrations of 50 nM each we found the signal to noise at high multiplexing levels was improved over the short multiplexing design. The ratio between chromosomes 21 and 6 was again as expected, increasing from 1∶1 to 10∶1 with the addition of more primer pairs targeting chromosome 21, and mean (n = 5) ratio values within upper and lower 95% confidence barriers (Figure 2B). In comparison with the small primers, the extended primers approach led to visibly more uniform PCR reaction efficiency, resulting in a smaller standard deviation of single molecule qPCR CT values in simplex reactions and higher signal to noise ratio in multiplexed reactions (Figure 3A and 3B respectively). Testing of this thermal protocol with the original short primer design resulted in poor performance (data not shown), indicating that the combination of new primer design, two-stage PCR, and the thermal protocol led to improved reaction performance.

#### Performance of assay designs in low volume chambers

**Figure 4:**
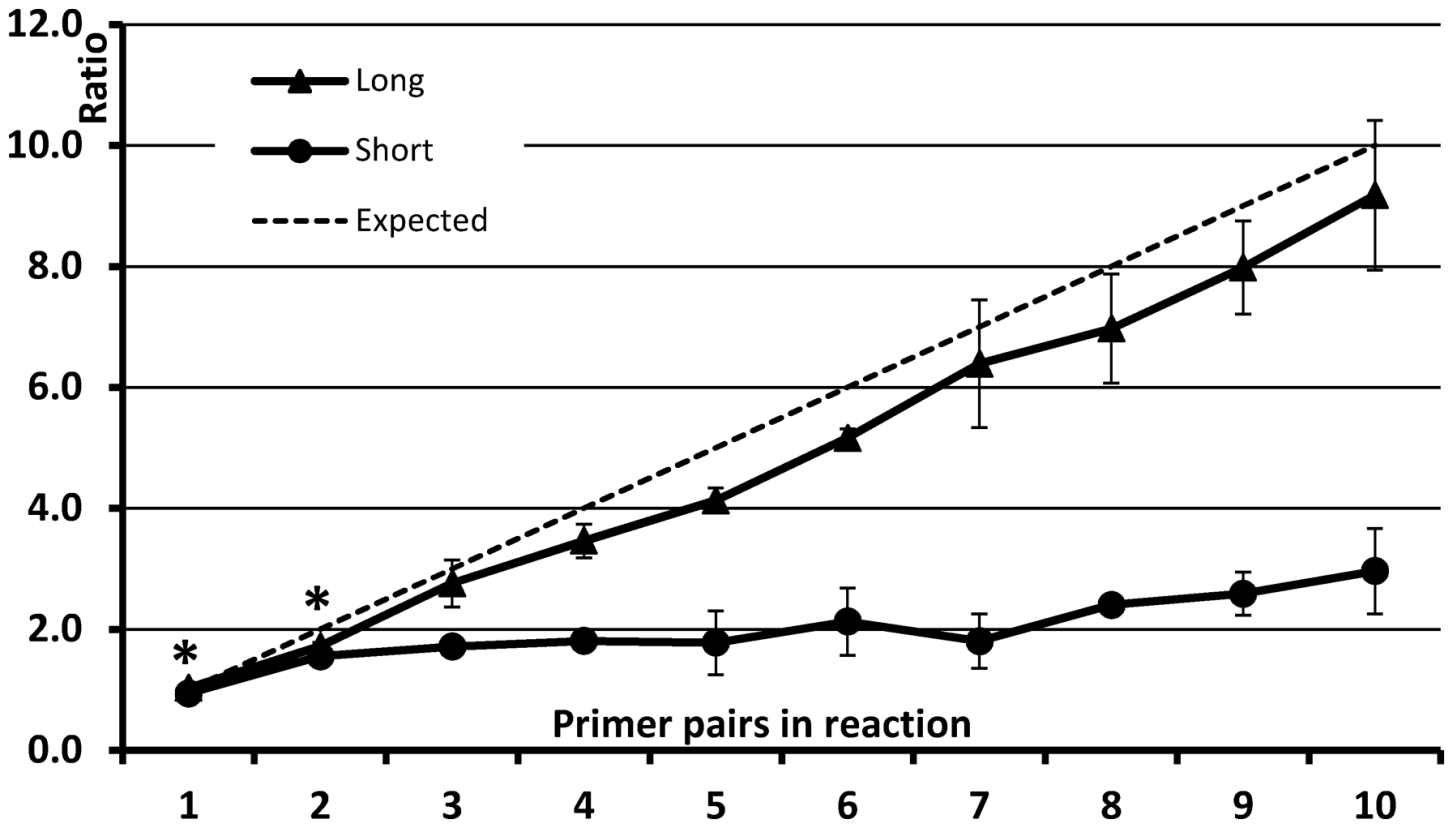
Effect of volume on dPCR reactions. Performance of same PCR mixes as in Figure 1B in 100 pL volumes. Two multiplexing approaches are compared: short primers and long primers plus a pair of universal primers. Multiplexing level in PCR reactions (horizontal axis) increases from 1× to 10×. Asterisks denote data points where difference between the two approaches was not significant (p = 0.05).

Although high-density dPCR formats provide improved dynamic range and precision, these formats require higher template concentrations and are also more sensitive to assay design since achieving good signal to noise is inherently more difficult due to reduced total PCR reagents and enhanced issues of evaporation during cycling. We evaluated the performance of both the short and long assay designs in high-density digital PCR arrays, with each sample being tested in arrays of 10,000 PCR reactions having 100-pL volumes. To minimize dehydration effects in small volumes [2] the following modifications were made to the thermal protocol: the hot start, denaturation and annealing times were all shortened, and the melting temperature and number of cycles were both decreased (Protocol 4, Table 2). At this volume the short primers failed to effectively detect targets at multiplexing levels above 3× (Figure 4). However, the long multiplex design again produced ratios close to expected values, likely due to a reduction in competing nonspecific amplification through lower total primer concentrations. Under these modified thermal conditions short primers were confirmed to perform well in 2 nL volumes (Figure S1), showing that the reaction volume accounts for the difference observed between the two strategies.

### Repetitive Simplex Strategy

In the two previous approaches we increased the multiplexing level by increasing the number of unique loci targeted per chromosome. However, we reasoned that in some cases it may be possible to increase multiplexing by exploiting naturally occurring repetitive sequences in the target molecules. To test this idea we sought to find and design an assay for chromosome 21 that targets chromosome-specific repetitive sequences with the following properties: a) are present at a defined and predictable copy number, b) highly conserved sequences without frequent polymorphisms in the assay design region, and c) unique to chromosome 21 only. We found a sequence that satisfied these criteria in a cluster of keratin genes located in the stretch of 45950000–46120000 bp of human chromosome 21 (GRCh37 human genome assembly). This region contains ten sequences that have a high degree of identity [14], allowing us to design a single PCR primer pair that uniquely targets all ten loci, generating amplicons that may be detected using a common fluorescent hydrolysis probe. As with the short and long multiplex designs, we performed dPCR analysis (Table 2, Protocol 3) with this assay and one targeting RNAse P (chr 6). These experiments generated ratios below the expected 10-fold enrichment of chromosome 21, with measured ratios of 7.3±0.8 (n = 10). We hypothesized that the observed lower than expected ratio could be explained by incomplete fragmentaion of the targeted loci; the mean distance between the targeted keratin genes on chromosome 21 is 17.5 Kbp. To resolve this discrepancy we pre-treating genomic DNA with the restriction enzyme BglII that has recognition sites between all ten target loci and repeated the dPCR analysis. Following this treatment measured ratios of chromosome 21 to chromosome 6 ratio were found to be 9.5±0.6 (n = 10), matching the expected ratio of 10∶1 (Figure S2). This result highlights that fragmentation is an important consideration for accurate dPCR analysis of multiple loci that are in close proximity. For loci that are separated by greater than 100 kilobases sufficient fragmentation is likely achieved during sample handling and shearing. We further note that clinical cell free DNA samples are typically of low molecular weight so that additional shearing is not required; however in such cases the design of short amplicons may be important for efficient detection.

## Discussion

We have presented and demonstrated three strategies for designing dPCR assays using Multiplex Template Sampling and show that this approach may be used to increase the effective copy number of large template molecules by 10-fold without compromising precision and accuracy. This approach provides a means to increase the precision of DNA copy number analysis and to facilitate sample preparation for clinical testing. We note that this approach is directly analogous to measuring small copy number variations by shotgun sequencing [8], [15], or targeted PCR followed by high-throughput sequencing [16], but presents potential advantages for clinical use, including measurement speed and cost. Commercially available dPCR platforms should allow for analysis of a sample in a few hours and at a cost comparable to existing diagnostic testing using RT-qPCR. In addition, interpretation of dPCR results is straightforward and does not require bioinformatic analysis. We note that the speed and simplicity of dPCR comes at the cost of much less information content as compared to sequencing. Although simultaneous non-quantitative amplification of multiple genomic loci using multiplexed PCR is well-established for sequencing or genotyping purposes [17], [18], [19], and most recently duplexing of dPCR was shown to increase precision [20], the use of high-level multiplexing of dPCR for accurate quantification has not been previously reported. Although we have demonstrated here the 10-fold multiplexing of loci on a single chromosome, accurate determination of allelic ratios in clinical samples would require extension of this method for multiplexed sampling of a reference chromosome, or the multiplexed targeting of multiple loci on different chromosomes.

Here we present three assay design strategies and protocols that effectively multiplex dPCR up to ten-fold without compromising performance. Although the ultimate multiplexing level has not been determined, this study provides some guidance for future design and testing. We found that the number of primer pairs in the PCR mix, and hence the degree of nonspecific amplification, is the main limitation for direct multiplexing approach when using standard ∼20 bp primers. Further, we observe that multiplexed assay performance is dependent on the volume of dPCR reactions. Specifically, multiplexed assays used in nanoliter volume dPCR failed at multiplexing levels higher than 4-plex when transferred to pL volume reactions. This indicates that assays should be optimized for a specific dPCR format, and also shows that larger volume formats may have advantages for multiplexing. We further show that small volume multiplexing is improved using a modular primer structure where one module functions at elevated temperature to recognize the specific loci, and the second is used later to develop the fluorescent signal needed for detection. This strategy works by reducing the concentraton of unique primers in order to minimize nonspecific amplification and was found to improve both the quality of fluorescent signal and the variation in Ct values (Figure 2, A). In comparison with the multiplexing approach using short primers, modular primers allowed us to lower concentration of individual primers by three times resulting in significantly lower total primer concentration (3.1 µM vs. 5.5 µM at 10× multiplexing). As result, the fluorescent signal in these reactions has a higher signal to noise ratio (Figure 2, B) and suggests that higher multiplexing levels are readily attainable. The downside of modular type of primers is their higher synthesis and purification cost. Although not tested here, we believe these multiplexing methods can be further improved by implementing multiplex primer design strategies that minimize interactions [21]. In addition we show that in some cases multiplexing can be effectively resolved by using primer pairs that each target more than one chromosome-specific loci. This approach requires extensive bioinformatics analysis and is not universally applicable since specific repeats may not be available for all targets of interest. Nevertheless, in cases where it is possible, this approach is preferred for both simplicity and performance.

## Supporting Information

Figure S1
**Effect of reaction volume on dPCR reaction counts.** Short multiplexed primer pairs (**A**) failed to perform as expected in low (100 pL) reaction volume at multiplexing levels above 2x (**-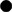-**). The same primer pairs returned expected counts at all multiplexed levels up to 10x in reaction volume of 2 nL (**-▴-**). Long multiplexed primer pairs (**B**) perform as expected, or close to expected in low (100 pL, **-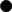-**) and big (2 nL, **-▴-**) reaction volumes at all multiplexing levels.(TIF)

Figure S2 **Effect of restriction digest on dPCR reaction counts in repetitive simplex strategy.** BglII restriction enzyme cuts genomic DNA between all 10 copies of keratin genes. Additional DNA fragmentation with this enzyme is necessary due to short distances between these genes. Ratio to the reference gene is shown.
(TIF)
