# Supplementary figures and images for: Methods for Multiplex Template Sampling in Digital PCR Assays
Source: PLoS One. 2014 May 22;9(5):e98341. doi: 10.1371/journal.pone.0098341 (PMC4031183; doi:10.1371/journal.pone.0098341)

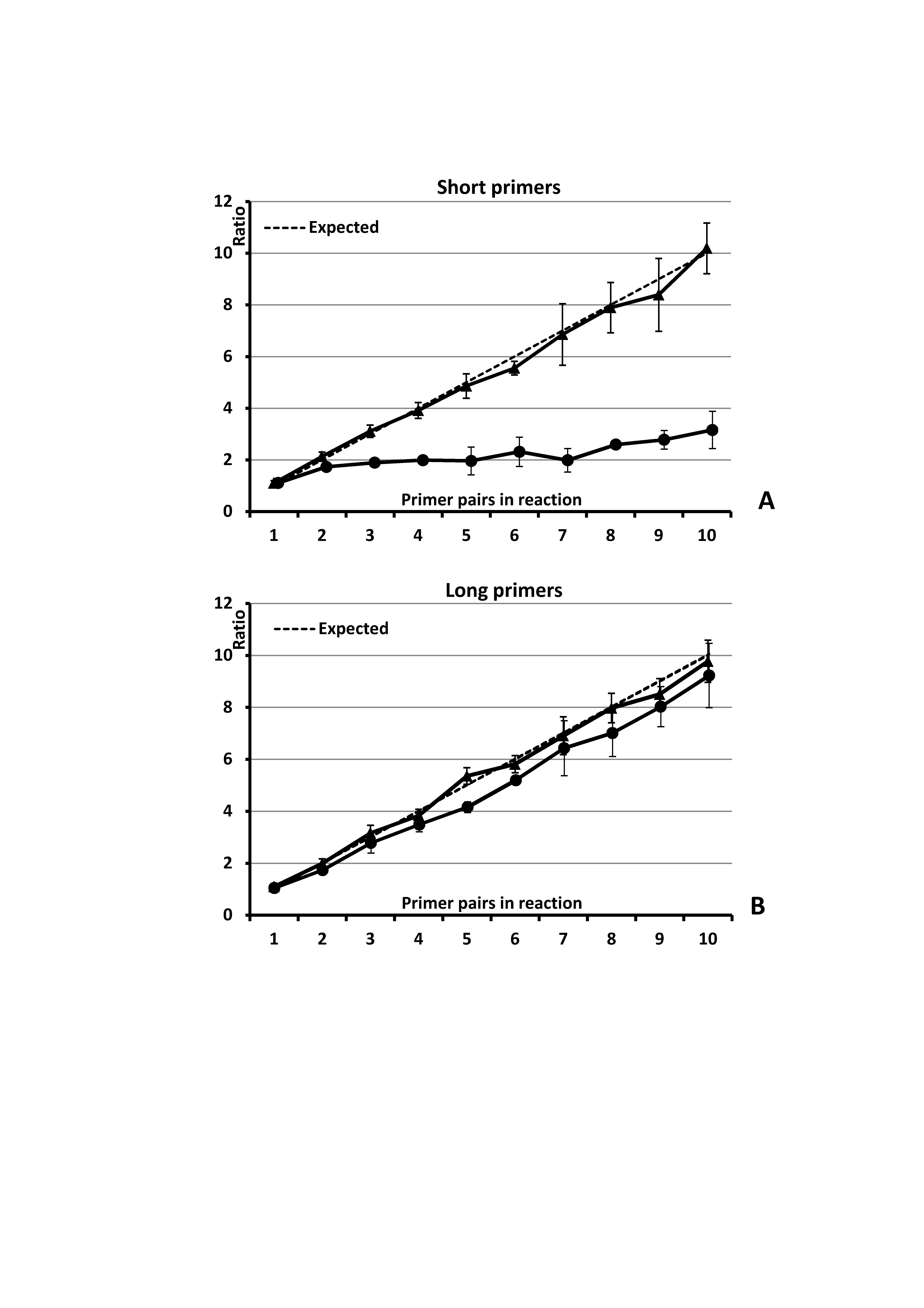

Supplement: Figure S1 — Effect of reaction volume on dPCR reaction counts. Short multiplexed primer pairs (A) failed to perform as expected in low (100 pL) reaction volume at multiplexing levels above 2x (--). The same primer pairs returned expected counts at all multiplexed levels up to 10x in reaction volume of 2 nL (-▴-). Long multiplexed primer pairs (B) perform as expected, or close to expected in low (100 pL, --) and big (2 nL, -▴-) reaction volumes at all multiplexing levels. (TIF) [file pone.0098341.s001.tif]

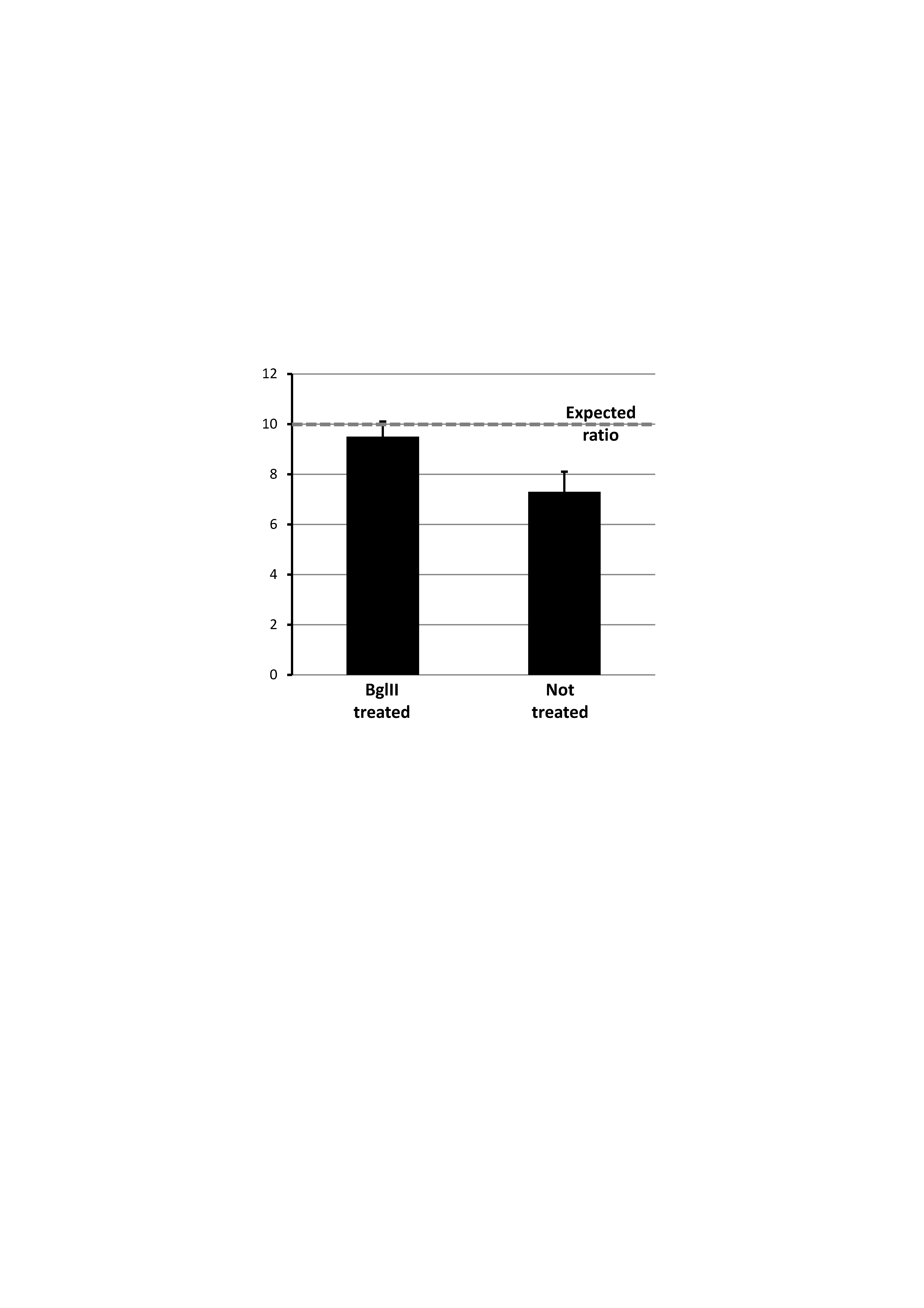

Supplement: Figure S2 — Effect of restriction digest on dPCR reaction counts in repetitive simplex strategy. BglII restriction enzyme cuts genomic DNA between all 10 copies of keratin genes. Additional DNA fragmentation with this enzyme is necessary due to short distances between these genes. Ratio to the reference gene is shown. (TIF) [file pone.0098341.s002.tif]
